# Microfluidic-Based Approaches for Foodborne Pathogen Detection

**DOI:** 10.3390/microorganisms7100381

**Published:** 2019-09-23

**Authors:** Xihong Zhao, Mei Li, Yao Liu

**Affiliations:** 1 Research Center for Environmental Ecology and Engineering, Key Laboratory for Green Chemical Process of Ministry of Education, Key Laboratory for Hubei Novel Reactor & Green Chemical Technology, School of Environmental Ecology and Biological Engineering, Wuhan Institute of Technology, Wuhan 430205, China; 13297902335@163.com; 2 School of Pharmacy and Food Science, Zhuhai College of Jilin University, Zhuhai 519041, China

**Keywords:** foodborne pathogens, microfluidic chip, rapid detection, food safety, biosensors

## Abstract

Food safety is of obvious importance, but there are frequent problems caused by foodborne pathogens that threaten the safety and health of human beings worldwide. Although the most classic method for detecting bacteria is the plate counting method, it takes almost three to seven days to get the bacterial results for the detection. Additionally, there are many existing technologies for accurate determination of pathogens, such as polymerase chain reaction (PCR), enzyme linked immunosorbent assay (ELISA), or loop-mediated isothermal amplification (LAMP), but they are not suitable for timely and rapid on-site detection due to time-consuming pretreatment, complex operations and false positive results. Therefore, an urgent goal remains to determine how to quickly and effectively prevent and control the occurrence of foodborne diseases that are harmful to humans. As an alternative, microfluidic devices with miniaturization, portability and low cost have been introduced for pathogen detection. In particular, the use of microfluidic technologies is a promising direction of research for this purpose. Herein, this article systematically reviews the use of microfluidic technology for the rapid and sensitive detection of foodborne pathogens. First, microfluidic technology is introduced, including the basic concepts, background, and the pros and cons of different starting materials for specific applications. Next, the applications and problems of microfluidics for the detection of pathogens are discussed. The current status and different applications of microfluidic-based technologies to distinguish and identify foodborne pathogens are described in detail. Finally, future trends of microfluidics in food safety are discussed to provide the necessary foundation for future research efforts.

## 1. Introduction

With the rapid development of the economy and the continuous improvement of living conditions, people today are paying more and more attention to health issues. At the same time, whether food is safe or not is also closely related to people’s health, therefore, it is also very important to ensure the safety of food. Unfortunately, people sometimes unconsciously eat some foods that are harmful to the body in their daily lives, for example, food contaminated by pathogens [1,2]. If people eat food containing foodborne pathogens such as *Staphylococcus aureus*, *Salmonella*, and *Escherichia coli* O157:H7, they may suffer vomiting or even death, triggering consumer panic [3]. According to the statistics of Parisi et al. a quarter of the world’s people are at higher risk of foodborne illnesses due to the current inefficient detection technology of bacteria, the imperfect food supervision system and high-speed economic development [4]. Overall, new strategies should be applied to improve food safety.

Foodborne illnesses are caused by pathogens or their toxins when they are contained in food or water. Pathogens causing foodborne illnesses include bacteria, viruses, fungi, and parasites [5]. For example, people infected by pathogenic *Escherichia coli* (*E. coli*) often experience severe diarrhea, and there are nearly 1.7 billion cases of diarrhoea every year in the world. More seriously, approximately 760,000 children under the age of five die each year from diarrhoeal diseases [6,7,8]. Most diseases are attributed to the common foodborne pathogens that include *Listeria monocytogenes*, *E. coli* O157:H7, *Staphylococcus aureus*, *Salmonella enterica*, *Bacillus cereus*, *Campylobacter jejunum*, and *Clostridium perfringens* [9]. Therefore, the effective detection of these pathogens is important.

At present, there are many methods to identify and detect pathogens, such as direct smear microscopy, nucleic acid hybridization, gene chip, polymerase chain reaction (PCR), gas chromatography and high performance liquid chromatography [10]. However, the most classic method is the plate cultivation method. However, this method requires three to seven days for bacterial culture, making it inappropriate for the rapid on-site detection of pathogens [11]. Additionally, PCR is also sometimes prone to false positive results due to DNA contamination [12,13]. Thus, to assess food safety, it is necessary to develop a rapid and simple method with high sensitivity, good reproducibility, and good on-site interpretation ability [14].

**Figure 1:**
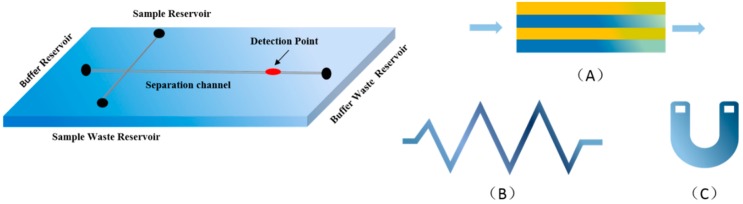
The structure of common microfluidic chip channel and variable styles of passive mixers. (**A**) Lamination; (**B**) Zigzag channels; (**C**) Serpentine.

For this reason, microfluidics with the advantages of portability, miniaturization and automation have been widely introduced to detect different substances in the fields of chemistry, biomedicine, optics and information science, such as dyes, bacteria or heavy metals [15,16]. Microfluidics are typically made of silicon, glass, quartz or thermoplastic materials. Then, micro-processing techniques are used to integrate micro-valves, micro-pumps, micro-mixers, micro-electrodes onto a micro/nanoscale chip to form a network-like system that can achieve pretreatment, mixing, reaction, separation or detection of the sample, which is not possible in traditional laboratories [17]. Microfluidics have several different types of basic mixer structures, as shown in Figure 1. For example, a microfluidic fluorescence quantitative PCR system with pneumatic valve and a tree structure was developed by using 3D printing technology. Due to its good temperature uniformity and thermal conductivity of PCR-based microfluidics, the rapid detection of hepatitis B virus nucleic acid in blood samples was realized in 50 min [18]. At present, the miniaturization, integration and automation of these devices combined with multiple processes have made microfluidic chips popular options for use in a wide range of fields, and the following are the applications and research status of microfluidics for bacterial detection.

In general, traditional microbial culture techniques require the use of tubes, culture dishes, multiwall plates and flasks, which makes the detection of bacteria more complicated. However, Wang et al. only combined a nano-dielectrophoretic enrichment-based microfluidic platform with surfaced-enhanced Raman scattering (SERS) to successfully and automatically monitor *Escherichia coli* O157:H7 in drinking water (the detection limited to single cell level) [19]. Wan et al. also developed a digital microfluidic system based on loop-mediated isothermal amplification (LAMP) for the detection of pathogen nucleic acids. In this experiment, only 1 µL of LAMP reaction sample that belong to purified *Trypanosoma cruzi* DNA was required, which reduced a 10-fold of reagent consumption compared to conventional LAMP. If the sample of LAMP is unknown, it also can be finished in 40 min with a detection limit of 10 copies/reverse. Moreover, the system can be thermally adjusted in real time, which is possible for the miniaturized, portable and on-site application in detecting bacteria in the future [20]. However, in order to reduce the costs and improve the portability of detection, high-performance materials such as paper-based microfluidic chips have been applied. Jokerst et al. designed a paper-based assay device for detecting *E. coli* O157:H7, *Salmonella typhimurium* and *Listeria monocytogenes*. The paper that was used for the preparation of the microfluidic system was wax printing on filter paper, which was achieved by measuring the color change of the response of the enzyme associated with the pathogen of interest to the chromogenic substrate. When combined with an enrichment procedure, the method allowed for an enrichment time of 12 h or less and was able to detect the bacteria in meat at a detection limit of 10 colony-forming units/cm^2^ [21].

As described above, microfluidic devices are simple, automated, and portable miniaturized systems that can perform functions more efficiently and conveniently than the common techniques such as PCR and LAMP [22,23]. Although there are some reviews on the application of microfluidic chip technology in food safety, their overall systematisms and integrity are not enough. This review not only describes the latest developments in integrated-microfluidic systems for detecting foodborne pathogens, but also discusses the most promising strategies to address current challenges for the faster and more accurate detection of foodborne pathogens by microfluidic chips. 

## 2. Microfluidic Chips

Microfluidic chips refer to the science and technology of systems that process or use very small volumes of liquids in channels with the dimensions of tens to hundreds of micrometres [24,25]. Microfluidics also are described as lab-on-a-chip (LOC) or miniaturized total analysis systems (μ-TAS), which integrate the sample preparation, reaction, separation, detection, and other basic operating units onto a centimeter-scale chip with a network of microchannels [26]. Microfluidics is an interdisciplinary field, including aspects of physics, chemistry, engineering, and biotechnology [24,27]. Due to the characteristics of electro-hydrodynamics with small size parameters and short detection times, electrodynamics, and thermal capillary phenomena, microfluidic devices have been developed to address specific scientific problems that are not able to be easily solved by traditional techniques [28,29].

Manz et al. first proposed the concept of a micro total analysis system (μ-TAS) [30]. In 1992, micro-electro-mechanical machining technology was used to etch micro-pipes on flat glass to prepare a chip capillary electrophoresis device, and the device realized the separation of fluorescently labeled amino acids and pioneered microfluidic chip technology [31]. In 1995, Woolley and Mathies successfully performed DNA sequencing using their own electrophoresis chip system, reading 150 bases in 540s with an accuracy rate of 97% [32]. Subsequently, Woolley et al. integrated PCR and capillary electrophoresis on a microfluidic chip, facilitating genetic analysis [33]. In 1998, Brahmasandra et al. (1998) used photolithography technology to fabricate a microfluidic chip that included a liquid sampler, a mixer, a positioning system, a temperature-controlled reaction chamber, an electrophoresis separation system, and a fluorescence detector system for DNA analysis [34]. In 2000, Anderson et al. developed a highly integrated chip that can be used to process a series of complex processes for multiple samples, and this device was applied for extracting concentrated nucleic acids from a liquid sample for microcrystalline chemical amplification, enzymatic reaction, hybridization, mixing, and measurement, allowing more than 60 consecutive operations of a dozen reactants [35]. 

**Figure 2:**
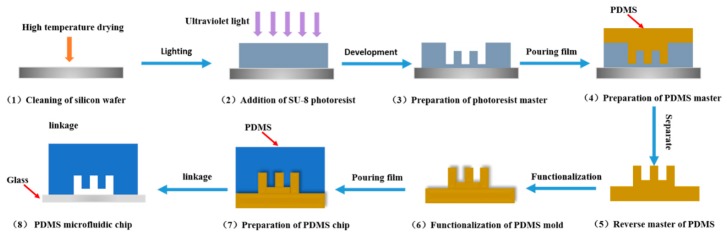
The preparation process of a polydimethylsiloxane (PDMS) microfluidic chip by the molding method.

Microfluidic devices are mainly operated by manipulating fluids in microfabricated channel and chamber structures. Additionally, microfluidics can be combined with diverse detection techniques including PCR, LAMP, mass spectroscopy, or fluorescence spectroscopy, for on-chip or after-chip detection of analytes [36,37,38,39]. Microfluidic chips are made of silicon, glass, quartz, organic polymer, and composite materials by micromachining technology. Figure 2 shows the preparation process of polydimethylsiloxane (PDMS) microfluidics. Recently, paper-based microfluidic chips with low cost, portability and easy operation have been developed in the food industry [40]. The selection of a certain material for a device is important for its functions. The different materials exploited for the fabrication of microfluidic chips and the advantages and disadvantages of these materials are listed in Table 1.

Compared to traditional methods such as PCR, enzyme-linked immunosorbent or DNA probes, microfluidic devices allow for a flexible combination of multiple operating units and overall controllability, so some steps such as sample pretreatment, mixing or reaction can be integrated into a single chip. Additionally, because the channel structure in the chip is micron-scale or even nanoscale, it has a high specific surface area, a high diffusion coefficient, and fast heat transfer, effectively accelerating the reaction in the channels and greatly shortening the overall analysis time [55,56]. For example, Zhang et al. developed a novel microfluidic liquid phase nucleic acid purification chip that can selectively separate DNA or RNA from 5000 μL to single cell bacterial cells. The sample volume is only 1 μL or 125 nL, which can be directly quantified by a chip in approximately 30 min. Thus, these small devices also require much lower amounts of reagents and samples, which greatly reduces the cost of detection and enables fast and low-cost detection [57]. Nevertheless, everything in the world has two sides. Without exception, microfluidic technology has its own disadvantages. For example, there is no skillful and mature technique for preparing a good microfluidic system and there is a lack of good and perfect preparation materials. Overall, the advantages of microfluidic technology confer promising potential for high-efficiency screening, environmental monitoring, clinical monitoring, on-site analysis, and DNA sequencing applications.

## 3. Sample Preparation in Microfluidics

### 3.1. For Single Component

For a single component, there is no special and complicated separation and purification treatment of the sample needed. However, how to improve the sensitivity, speed and accuracy of the detection component is particularly important. At present, the molecular technologies, such as PCR, first need to extract the DNA of the bacteria, and also add the required reagents by labour, which is extremely time-consuming and troublesome. In addition, if the concentration of the analyte does not reach a measurable level, it is also necessary to concentrate the bacterial DNA concentration multiple times [58]. Therefore, other methods, such as optical analysis, fluorescence detection or electrochemical analysis, can avoid the pretreatment of samples and achieve automated, simple and rapid bacterial detection.

PCR is routinely applied to detect some components in food. Therefore, Tachibana et al. developed a new PCR-based microfluidic technique for the successful detection of 0.031 μg/μL of *E. coli* O157:H7 genomic DNA, which was completed in 18 min and provided a new platform for a rapid, simple and low-cost detection assay for this pathogen. However, there is still a need to further improve the bacterial pre-enrichment and DNA purification steps to lower the detection limit of *E. coli* O157:H7 in the integrated PCR system (10^3^ CFU/mL) [59]. Zhang et al. used magnetic silica beads and a special coaxial channel to optimize the detection of *E. coli* O157:H7. This special channel allows the improved separation and capture of the lysed DNA of *E. coli* O157:H7 using magnetic materials. With this modified system, *E. coli* was successfully detected by microfluidic PCR with a detection limit of only 12 CFU/mL [57].

To lower the cost and the difficulty of sample preparation, and increase the portability of testing, high-performance materials, such as paper-based microfluidic chips, are being developed to detect such pathogenic bacteria. Wang et al. proposed a paper-based impedance immunosensor for detecting *E. coli* O157:H7. Gold nanoparticles grew on the working electrode and anti-*E. coli* O157:H7 antibody immobilized on the paper electrode was used to capture the target bacteria, which changed the resistance of the reaction in different environments and successfully detected *E. coli* O157:H7 from ground beef (LOD of 1.5 × 10^4^ CFU/mL) and cucumber (LOD of 1.5×10^3^ CFU/mL) [60]. Moreover, there are also numerous introductions and research on paper-based microfluidics. Cate et al. reviewed the preparation, principles, and application of paper-based microfluidic chips [61]. Liu et al. also described recent developments, trends, and challenges of paper-based microfluidic chips for food safety applications [62]. Therefore, the detection process for pathogens is mainly to optimize the detection technology.

### 3.2. Complex Components in Food Matrix

To date, most techniques that are used to determine some experimental samples are relatively simple just for a single component. However, for practical use, complex samples and variability in environmental conditions may result in reduced sensitivity and specificity of microfluidic technology, so a device should be designed to analyze more complex samples, such as soil, sewage, or food samples [63]. Due to the physical and chemical properties of each component to be tested in the sample may not be much different than those of single components (such as the detection of different bacteria), it may be difficult to achieve simultaneous detection of multiple components. Additionally, the variety and content of other substances in complex food matrices may interfere with the detection and reduce the accuracy of the assay. For example, if the aim is to detect *E. coli* in food samples, there is definitely more than one kind of bacteria in this concentrated sample. Thus, in order to eliminate these interferences, some specific bio-recognition molecules can be integrated into the detection system. It is by increasing the concentration and specificity of the sample that makes it easier to detect the components from complex mixtures.

#### 3.2.1. Special Materials and Sampling Methods

The main cause of low reproducibility or the inability of microfluidic devices to detect analytes in complex food substrates is due to the concentration of analytes below the detection limit. Thus, the separation and enrichment of targets from a food matrix are needed to increase the efficiency for detecting analytes. The sample concentration can be improved using a variety of techniques such, as magnetic beads or filter membranes. Among them, the magnetic beads generally have superior paramagnetism, such as Fe_3_O_4_, which is able to separate from the sample to be tested with the help of a magnetic field and a rich surface-active group. The filtration membranes are usually made of a variety of ultra-high-performance polymers, which have acid and alkali resistance or oxidation resistance to achieve the separation and purification of the samples. Furthermore, since the reaction is performed in a microfluidic system, different injection methods may improve the concentration of the target [18,64].

Immunomagnetic separation (IMS) can be used to concentrate bacterial cells present at lower concentrations, but it is just suited to a small volume sample (e.g., 1 mL), which is far smaller than the large volume of enrichment culture (e.g., 250 mL). To address this issue, Ganesh et al. integrated IMS of bacterial cells into microfluidic devices for the preconcentration of 50 mL volume samples. PCR was then applied for the qualitative and quantitative detection of the *E. coli* O157:H7 in less than two hours. This platform decreased both the required sample volume and the overall time of the reaction [65]. Oh et al. combined loop-mediated isothermal amplification (LAMP) with a disk-shaped centrifugal microfluidic device to successfully detect four foodborne pathogens (*Escherichia coli* O157:H7, *Salmonella typhimurium*, *Vibrio parahaemolyticus* and *Listeria monocytogenes*) in contaminated milk samples with bacteria. The use of Eriochrome Black T (EBT) in the system allowed the colorimetric detection of the LAMP reaction, and this process enabled a fully automated detection of bacteria with a detection limit of 10 bacterial cell level in 65 min [66]. However, the colorimetric measurement of this platform is identified by the naked eye, which may cause some errors in the interpretation of the experimental results. For this reason, Sayad et al. utilized calcein as an indicator and combined it with LAMP for a genotypic analysis of eight strains of the foodborne pathogens *E. coli* O157:H7, *Salmonella* and *Vibrio cholerae*, for a total of 24 pathogenic bacteria being detected. The result of the colorimetric method was analyzed and transmitted to a smartphone using a developed electronic system that interfaced with bluetooth wireless technology in 60 min. This system avoids artificial subjective errors and achieves a fully automated, quick and on-site test [67].

The above experiments use special materials to detect bacteria, but filter membranes can also be used to increase the concentration of the target. Li et al. used a poly sulfone hollow-fiber membrane module to separate and concentrate bacterial cells from chicken homogenates in cross-flow microfiltration. This special microfluidic system can effectively recover 70% of the analytes in the mixture in 30-45 min, greatly improving the concentration of analytes and decreasing experimental time (approximately 6 h in the industry) [68]. However, special microfluidic injection channels can also be used. For example, Shu et al. integrated multiple PCR steps into microfluidics by preparing special continuous-flow channels. With this special device, the genes of *S. enterica*, *L. monocytogenes*, *E. coli* O157:H7, and *S. aureus* could be simultaneously amplified and detected from banana, milk, and sausage samples. The whole experiment required only 19 min, with a detection limit as low as 10^2^ copies/μL [69].

#### 3.2.2. Bio-Recognition Molecules

**Figure 3:**
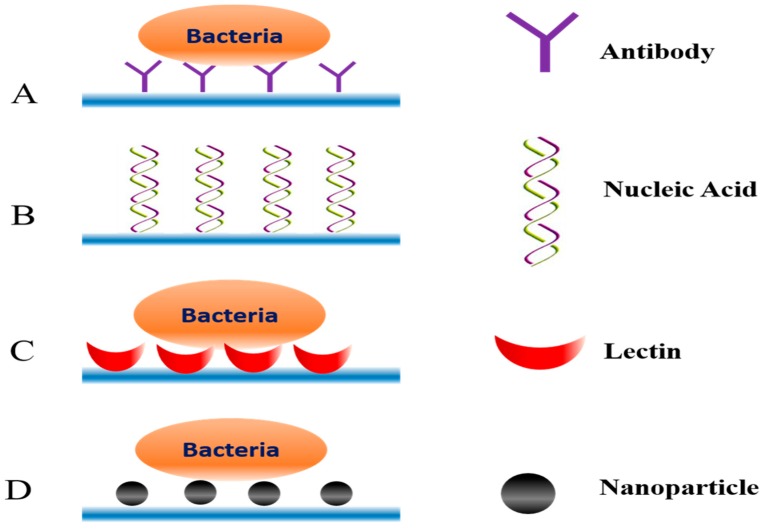
Schematic view of different bio-recognition elements in microfluidics. (**A**) Antibody; (**B**) Nucleic Acid; (**C**) Lectin; (**D**) Nanoparticle.

Even if the concentration of analytes can be increased, the detection of the target in the presence of some similar components is challenging. Therefore, some biomarkers capable of specifically recognizing the analyte are required to achieve the rapid and accurate detection of the target. As shown in Figure 3, there is the high specific interaction between some surface antigen biomarkers and recognition molecules.

Antibodies are one of the most common bio-recognition molecules. Savas et al. used a biosensor-conjugated antibody on gold nanoparticles to successfully detect *Salmonella* from human stool samples. The fully automated microfluidic electrochemical sensor allowed *Salmonella*, as low as 1 CFU/mL, to be sensitively and specifically detected in mixed samples by a specific reaction between the specific antibody and the antigen on the surface of the bacteria [70]. As an alternative to antibodies, aptamers are single-stranded nucleic acid molecules that are stable, easy to synthesize, and cheaper than antibodies. Aptamers can also specifically bind target molecules and can be modified with various fluorescent dyes or other labels. Wu et al. first separated and concentrated analytes from a mixed solution using the property of Fe_3_O_4_ magnetic nanoparticles. Next, according to the specificity of the aptamer of different bacteria, color-changing upconverting nanoparticles conjugated with different aptamers were used as a signal probe to detect three corresponding pathogenic bacteria. The color change of the multi-color upconverting nanoparticle composite indicated whether the bacteria existed in the mixture to achieve simultaneous, sensitive and selective detection [71].

Lectins can also be used as a bio-recognition molecule. Kang et al. studied different sizes of magnetic nanoparticles coated with lectins for the capture of pathogenic bacteria from mixed solutions. The result showed that magnetic nanoparticles with a radius of 250 nm were the most effective method for separating and detecting *S. aureus* in a mixed solution (10^2^ CFU/ mL) [72]. Another study used concanavalin A (ConA), a mannose/glucose-binding lectin that can be used to recognize lipopolysaccharides exposed to bacterial surfaces. Dao et al. combined ConA-functionalized microfluidic chips with LAMP to capture and enrich *Salmonella typhimurium* in urine samples (10 mL). Through this integrated system, the label-free, fast and real-time detection of *Salmonella typhimurium* with a concentration as low as 5 CFU/mL was completed in 100 min [73].

## 4. Application of Microfluidic Combined with Different Technologies

Currently, traditional technologies, such as PCR, ELISA and LAMP, are accurate and effective, but they may be costly and complicated [74,75]. Furthermore, for food or other complex environmental samples, the acquisition of the analytes may be difficult or it may be challenging to completely integrate the separation and detection processes in a single microfluidic chip [76]. In particular, if the physical and chemical properties of each component to be tested in the sample are similar, it may be difficult to simultaneously distinguish and detect various substances. The future work should aim to decrease pre-processing or to combine pre-processing steps with detection for the analysis of foodborne pathogens. The successful application mainly depends on high efficiency, high speed, and the automation of microfluidic technology, combined with different technologies, such as electrochemical biosensors, optical biosensors, immunoassays and nucleic acid-based methods [77].

### 4.1. Biosensor-Based Microfluidics for the Detection of Foodborne Pathogens

Biosensors are developed based on knowledge from the disciplines of biology, chemistry, physics, medicine, and electronic technology. A biosensor is sensitive to biological substances and can convert signals, such as the concentration and activity of analytes, into electrical signals for rapid detection [78,79,80]. Safavieh et al. used a microfluidic electrochemical biosensor that combined with LAMP for the detection and quantification of *E. coli*. There is no need of probe immobilization, and bacterial detection can be done in a single chamber without DNA extraction and purification steps. This experiment can detect and quantify bacteria to 24 CFU/mL and 8.6 fg/μL of DNA within 60 min [81]. This shows the use of biosensors in microfluidic chips may provide integrated systems with improved sensitivity and rapid and on-line detection. Biosensors include both the optical biosensors and electrochemical biosensors [82]. 

#### 4.1.1. Microfluidic Chips with Optical Detection

##### Surface Plasmon Resonance (SPR) Biosensors

**Figure 4:**
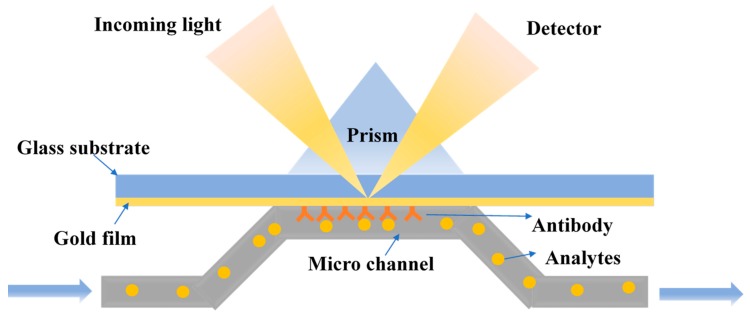
The principle of surface plasmon resonance detection.

Surface plasmon resonance (SPR) is a high-sensitivity and real-time spectral analysis technique that measures the change in the refractive index of a surface material on a metal film. The application of SPR for the detection of analytes is shown in Figure 4. The advantage of SPR is that the object tested is label-free, and the method is easy and quick, allowing dynamic and real-time monitoring of the reaction [83,84]. At present, SPR has been used to detect pathogenic bacteria, allergens, and toxins [85].

Zordan et al. designed a hybrid microfluidic biochip for the detection of pathogens using SPR combined with fluorescence imaging. An array of gold spots was included in the microfluidic system to specifically capture the specific pathogens. A closed polydimethylsiloxane (PDMS) microfluidic flow chamber was used to transport and magnetically concentrate the sample to be tested. SPR and fluorescence were then used for the successful detection of *E. coli* O157:H7 [86]. The Zordan’s group also developed a biosensor array chip to specifically detect the presence of different pathogens. In this design, the PDMS microfluidic system allowed SPR and fluorescence imaging for simultaneous, rapid, label-free, real-time and multiple detection of foodborne pathogens. Furthermore, the functionalized magnetic particles were applied to a hybrid microfluidic biochip [87]. Tokel et al. prepared a portable, low cost and multiplexed microfluidic system that used SPR to detect and quantify *E. coli* and *S. aureus*. As a result, 100 μL of *E. coli* or *S. aureus* in phosphate buffered saline and peritoneal dialysis solution at a concentration of 10^5^ to 3.2 × 10^7^ CFU/mL can be reliably and specifically detected within 20 min [88].

##### Optical Fibre Biosensors

Fiber-optic biosensors can selectively interact with a specific biosensor (i.e., antigen-antibody or enzymes), resulting in the production of biological or chemical information that can be converted into a transmitted light signal captured by the optical fiber, with varying light intensities, light amplitude, or phases [89]. An ideal sensor has good selectivity and high sensitivity for bacterial pathogens, pesticides, and toxins [90,91]. However, the spectra generated by the complexes or products formed in the experiments are similar, so the fibers are unable to be easily distinguished and detected. Therefore, the indicators or labels, such as enzymes, fluorescent substances, acid-based indicators, and lanthanide complexes are often used. Instead, optical fiber biosensors are mostly used in conjunction with various spectroscopy techniques such as absorption, fluorescence, or surface enhanced Raman spectroscopy (SERS) to improve sensitivity.

The Raman signal from molecules located near a nano-structured metallic surface and excited by visible light can be strongly enhanced, a process known as surface enhanced Raman scattering (SERS). SERS is widely used in the detection of foodborne pathogens. Li et al. invented a microfluidic chip with an integrated nanoporous gold disk array, a highly effective SERS substrate. The integrated system has an order of magnitude of a larger surface area than its projected disk area, corresponding to a great improvement of the Raman signal. Rhodamine was used to test the performance of the microfluidic device, showing excellent and rapid detection [92]. Mungroo et al. developed a microfluidic device with silver nanoparticles to improve the detection of pathogenic bacteria. The data analysis included homometric, principle component, and linear discriminant analyses. This platform allowed the detection and discrimination of multiple major foodborne pathogens: *E. coli* O157:H7, *Salmonella*, *S. enteritidis*, *P. aeruginosa*, *L. monocytogenes*, and *L. innocua* [93].

Gilli et al. designed a disposable plastic sensing device that utilized a total internal reflection fluorescence optical. There is no interference caused by non-specific binding or noise, and the microfluidic chip is connected with automated and sensitive customized software to realize the multiplex detection of the different targets [94].

#### 4.1.2. Microfluidic Chip with Electrochemical Detection

Electrochemical biosensors use electrodes as conversion elements and immobilize bio-sensitive substances including antigens, antibodies, or enzymes onto the electrode to detect target molecules by specific bio-recognition and antigen interaction [95]. The above reactions can be transformed into electrical signals, such as capacitance, current, potential, or conductivity, to achieve the qualitative or quantitative detection of analytes, resulting in powerful tools for the detection of biological samples [96,97,98].

Tan et al. developed a stable PDMS microfluidic device with an impedance immunosensor by grafting modified silane and an antibody on nanoporous membranes for the specific measurement of *E. coli* O157:H7 and *S. aureus*. The difference between these bacteria was expressed by monitoring the amplitude change of the impedance spectrum before and after the bacteria captured by complimentary antibodies on the nanoporous alumina membrane, which achieved a rapid and sensitive bacterial assay of 10^2^ CFU/mL in 2 h [46]. Chen et al. developed a fast, sensitive and complex microfluidic device that integrated electrochemical impedance analysis and urease catalysis to measure *Listeria*. The bacteria cells, the modified magnetic nanoparticles (MNPs) with anti-*Listeria* monoclonal antibodies, anti-*Listeria* polyclonal antibodies, and the urease modified gold nanoparticles (AuNPs), were incubated in an integrated microfluidic chip with active mixing to form MNP-*Listeria*-AuNP-urease sandwich complexes. Through this platform, *Listeria* can be detected as low as 1.6 × 10^2^ CFU/mL in one hour [99]. 

Overall, the use of online, automated, and sensitive microfluidic impedance biosensors for bacterial separation and detection is promising. To improve the effectiveness of these systems, Liu et al. integrated dielectrophoresis and electrochemical impedance into microfluidics for in-situ impedance detection of bacteria. The dielectrophoresis technique was applied to enrich trace bacteria. The microarray electrode microfluidic chips can specifically detect bacteria from microsystems. The detection limit of *E. coli* O157:H7 in this device was 5 x 10^4^ CFU/mL in 6 min [100]. This integrated microfluidic analysis microsystem is the first step for the rapid real-time in situ detection of bacteria.

The above devices are impedance-based for the detection of foodborne pathogens. In addition, there are voltametry-based microfluidics. Safavieh et al. used LAMP in a microfluidic system for the quantitative detection of *E. coli* O157:H7 and *S. aureus* using the linear sweep voltametry method. The foodborne pathogens with a detection limit of 48 CFU/mL were detected in 35 min. Unlike other electrochemical techniques, this method does not require a complex probe immobilization process, and bacterial detection can be performed in the chamber structure without the need for DNA extraction and purification steps [81].

### 4.2. Immunoassay-Based Microfluidics for the Detection of Foodborne Pathogens

**Figure 5:**
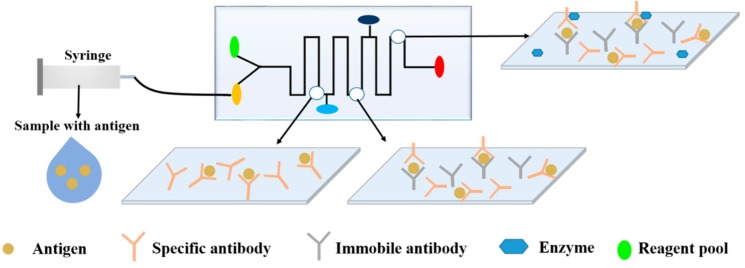
The basic principles of immunoassay-based microfluidics for the detection of foodborne pathogens.

The immunological methods offer high specificity, high sensitivity, and high analytical capacity based on the specific reaction between the antigen and antibody to form a complex, as shown in Figure 5. The immunological approaches have been used to detect bacteria, viruses, fungi, various toxins, parasites, proteins, hormones, other physiologically active substances, drug residues, and antibiotics [101]. The determination of pathogenic bacteria by immunological methods alone is prone to cross-contamination risks and negative results, and requires trained personnel. However, when combined with microfluidic technology and immunoassays, specific antigen-antibody reactions can enhance the specificity and sensitivity of microfluidic analysis. Additionally, the use of microfluidics is rapid, has low-consumption, and automated compared to traditional immunology techniques, such as ELISA, lateral flow assays (LFAs), or radioimmunoassays (RIAs), which may require long detection times, expensive reagents, or complicated procedures [102].

#### 4.2.1. Enzyme-Linked Immunosorbent Assay (ELISA)

In ELISA, a known antigen or antibody on the surface of a solid phase carrier (polystyrene microplate) is bound in an enzyme-labeled antigen-antibody reaction, and any free components in the liquid phase are washed away. This method has been applied to the effective and specific detection of pathogenic bacteria [103,104]. 

Thaitrong et al. designed a microfluidic sandwich ELISA for the rapid determination of plant pathogens. The microfluidic concentrator was fabricated using a microchannel, and the all reactions were in a microfluidic channel with the help of capillary force to drive the flow of the reactants [104]. Compared to traditional methods, this microfluidic system is faster, more portable, energy-efficient, and protected against sample contamination, providing a new approach for the detection of pathogens [92]. In a similar device by Wu et al. analytes were concentrated by mixing iron particles with PDMS to form an electromagnetically-driven microdevice that could be controlled by the application of a magnetic field [105], as described by Yanagisawa and Dutta [106].

#### 4.2.2. Immunomagnetic Fluorescence Assay (IMS)

The IMS assay is also based on the reaction between the antigen and antibody. When the IMS assay combined with microfluidic-based technology, the performance of the IMS assay can be more highly specific and sensitive, fast, and convenient. Zhang et al. connected an optical fiber spectrometer with a microfluidic device to achieve the rapid and sensitive detection of avian influenza virus. The integrated device allowed the immunomagnetic capture, concentration, and fluorescence detection of foodborne pathogens [107]. Similarly, Kanayeva et al. combined magnetic nanoparticles, a microfluidic chip, and an interdigitated microelectrode to integrate an impedance immunosensor for the efficient separation and sensitive detection of *L. monocytogenes* [108].

There are several other microfluidic based immunological methods, such as lateral flow assays (LFAs) [109,110] and RIAs [111]. Although the combination of immunology and microfluidics has greatly improved its performance, further improvement is possible. For example, non-specific binding is a problem and increased objectivity is required for result interpretation, as it can lead to wrong results and affect later experiments [112]. 

### 4.3. Nucleic Acid-Based Microfluidics for the Detection of Foodborne Pathogens

Nucleic acid-based methods can be used to detect a certain sequence of DNA or RNA from pathogens, using capture and detector probes (short DNA or RNA sequences). These methods can provide more specific and accurate results than the above methods. The integration of nucleic acid-based detection technology in microfluidic devices has been widely applied in various fields due to the small-volume sample requirement, fast detection time, and simple sample processing, especially for the detection of foodborne pathogens [56,113]. Nucleic acid-based detection methods include PCR, LAMP, and recombinase polymerase amplification (RPA). 

#### 4.3.1. Polymerase Chain Reaction (PCR)

Ganesh et al. designed an integrated microfluidic PCR system consisting of two main components: A preconcentration chamber for the immunomagnetic separation of bacterial and a PCR chamber for DNA amplification. Further, *E. coli* O157:H7 with the detection limit of 10^3^ CFU/mL was successfully detected by the integrated system [65]. Zhang and Wang developed an integrated microfluidic platform with silica superparamagnetic particle-based solid phase extraction for cell lysis, DNA binding, washing, elution, and PCR on a single platform [114]. The preparation, principle, and usage of specific PCR-based microfluidic chips have been described [58,59].

#### 4.3.2. Multiplex PCR

Zhang et al. reported a flow-based multiplex PCR microfluidic system capable of high-throughput and rapid DNA amplification to detect foodborne pathogens. The system consisted of four reaction channels to simultaneously detect *L. monocytogenes*, *E. coli* O 157:H7, and *S. enterica* from food samples. Multiplex PCR with a special injection device of oscillatory-flow used only 5μL of the sample and the reaction can be completed in 13 min, being one sixth of the time required for conventional PCR (70 min) [115]. Similarly, Shu et al. prepared a segmented continuous-flow multiplex PCR on a special spiral channel microfluidic device that consists of a disposable polytetrafluoroethylene capillary microchannel coiled on three isothermal blocks. The microfluidic device rapidly identified a variety of foodborne pathogens, including *S. enterica*, *L. monocytogenes*, *E. coli* O157:H7 and *S. aureus*. After optimizing the parameters, their genomic DNA of four bacteria were amplified simultaneously at 19 min with a minimum detection limit of 10^2^ copies/μL [69].

#### 4.3.3. Loop-Mediated Isothermal Amplification (LAMP)

Compared with enzyme-linked immunosorbent assays, LAMP is a rapid and specific method for nucleic acid amplification. LAMP does not require thermal denaturation, temperature cycling, electrophoresis, or ultraviolet detection, and it shows better sensitivity, specificity, cost and detection range than PCR. Additionally, LAMP does not require a complex temperature gradient regulation for high-throughput rapid detection.

Tourlousse et al. developed a cheap, portable, easy-to-use, single use polymeric microfluidic chip for the quantitative detection of different pathogens by isothermal nucleic acid amplification. The microfluidic chips were able to rapidly and quantitatively detect bacteria DNA of 10–100 genomes/μL in 20 min [116]. Uddin et al. prepared a rapid, automatic and novel microfluidic compact disk platform combined with LAMP and a color sensor for the sensitive detection of different DNA concentrations for *Salmonella*. Furthermore, a disk platform can achieve a simultaneous detection of multiple sets of samples [117]. For simultaneous detection of complex samples, Sun et al. described an eight-chamber microfluidic chip that takes advantage of magnetic bead-based sample preparation and LAMP for the rapid quantitative detection of *Salmonella* in food samples. The system can measure *Salmonella* at concentrations of 50 cells per test within 40 min for rapid on-site screening of foodborne pathogens [118].

However, nucleic acid-based microfluidics for the detection of foodborne pathogens include RPA, a nucleic acid detection technology that allows for single-molecule nucleic acid detection at room temperature within 15 min. This technology is truly portable and fast, with acid-based detection for analytes and low requirements for hardware equipment [119,120]. Other methods for pathogen detection include nuclear acid sequence-based amplification (NASBA) and nuclear acid sequence-based amplification (HAD) [121,122,123].

## 5. Challenges and Opportunities

Microfluidics integrates the functions of a full laboratory into a single device, including sampling, dilution, reagent addition, reaction, separation, and detection. The potential applications of microfluidics in the food industry include the detection of foodborne pathogens, but also the detection of pesticide residues, heavy metals, or food additives. Microfluidic devices require lower consumption of reagents, and provide faster screening with shorter reaction times and lower costs. Thus, microfluidic technology provides promising approaches to solve key and complex problems in food safety.

However, the application of microfluidics based on different technologies for the detection of foodborne pathogens is still in its infancy. Although some special materials and bio-recognition molecules can be used to improve the detection of targets in actual samples, there may be some non-specific binding that can influence the results of the experiment. The severity of this problem, based on the composition of the food samples and the variation of the sample pretreatment processes, is described by Li. et al. [124]. However, current analytical systems are relatively immature, so the detection of pathogenic bacteria is not yet precise [72]. Furthermore, a major challenge to be overcome is that existing microfluidic systems are complex or expensive to easily integrate into a functional system, and such ease of integration is required for convenient use in food safety. However, there are great expectations for further innovation and development of highly efficient microfluidic technologies for measuring pathogenic bacteria.

## 6. Conclusions

Food safety is closely related to human health, therefore, powerful, sensitive and effective tools are needed to ensure food safety, such as the detection of foodborne pathogens. The high selectivity, sensitivity, and efficiency of microfluidic technology can employ these devices to replace some traditional labor-intensive and slow-culture methods for the detection of pathogens in foods. However, capturing effective pathogens from complex food samples for high-throughput multiplex analysis remains inefficient. Consequently, when some traditional methods are combined with microfluidic technologies that can be more effective, the preconcentration and sample preparation steps are typically improved and simplified. This article described the incorporation of rapid detection techniques such as SPR, ELISA, PCR, and LAMP in microfluidic devices for improving the detection efficiency of foodborne pathogens.

Nevertheless, the technologies for preparing microfluidic devices and integrating microfluidics with other detection technologies are imperfect and are in the initial stages of industrialization. Therefore, further exploration and research is needed to expand the application of microfluidic technology in different industries. The authors are confident that microfluidics will be more broadly applied in multiple fields, once these problems are addressed in future studies. 

## Figures and Tables

**Table 1:** The application of a microfluidic system made of different materials.

Material Type	Classification	Representative	Methods of Preparation	Advantages	Disadvantages	Application	References
**organic material**	**-------**	glass/quartz	photolithography and etching techniques	cheap and easy to obtain, reusable, good light transmission and electroosmosis, good electrical insulation and corrosion resistance	complex manufacturing process, time-consuming and high cost, fragile	gas chromatography and capillary electrophoresis (CE) and electrochemical detection, organic synthesis and droplet formation, PCR	[41,42]
	silicon material	silicon/silicon dioxide	etching techniques	mature process, good thermal stability and inertness.	high cost of materials, opaque, brittle, poor electrical insulation, and low adhesion coefficient	organic synthesis and droplet formation, PCR and CE	[43,44]
	elastomers	polydimethylsiloxane (PDMS)	molding and soft lithography	Low cost and easy to use, non-toxic and transparent, excellent chemical inertness and light transmission	Incompatibility of organic solvents and poor pressure resistance, low thermal conductivity and immature processing technology	protein crystallization and bioculture, PCR	[45,46]
**Polymer materials**	thermosets	SU-8 photoresist and polyimide	photopolymerization and casting	High resistance of temperature and most solvents, transparent and reusable	high cost of materials	CE, organic synthesis and droplet formation, PCR	[47,48]
	thermoplastics	poly (methyl methacrylate (PMMA) polystyrene (PS) and polycarbonate (PC)	hot embossing and laser ablation	good electrical insulation and light transmission, low cost and easy to use, simple preparation and high precision	Non-breathable, high-cost preparation equipment and rough process	CE and PCR, droplet formation	[49,50]
	perfluoropolymers	perfluoroalkoxy (PFA) and fluorinated ethylene propylene	photolithography	Good inertness and antifouling properties, transparent and soft	poor adhesion	environmental monitoring and food analysis	[51]
**Special materials**	hydrogels	polyvinyl alcohol (PVA)	photopolymerization, casting	high permeability and controllable aperture, allowing small molecules or even biological particles to diffuse, and biocompatible	difficult to store	3D bioculture	[52]
	ceramics	polysiloxane	soft lithography and laser ablation	high resistance of temperature and pressure	poor light transmission, fragile	suitable for applications under harsh conditions	[53]
	paper	analysis filter paper	photolithography and printing	high permeability and low cost, portable and easy to use	easy to damage and disposable	bioculture	[54]
